# Supplementary material for: Fatty Acid Synthase as Interacting Anticancer Target of the Terpenoid Myrianthic Acid Disclosed by MS-Based Proteomics Approaches
Source: Int J Mol Sci. 2024 May 29;25(11):5918. doi: 10.3390/ijms25115918 (PMC11172900; doi:10.3390/ijms25115918)
Supplement: Supplementary file 1 [file ijms-25-05918-s001.zip › ijms-3004605-supplementary.pdf]

Fatty Acid Synthase as Interacting Anticancer Target of the Terpenoid Myrianthic Acid Disclosed by MS-Based Proteomics Approaches

Alessandra Capuano [1,2], Gilda D'Urso [1,*], Erica Gazzillo [1,2], Gianluigi Lauro [1], Maria Giovanna Chini [3,*], Maria Valeria D'Auria [4], Maria Grazia Ferraro [5], Federica Iazzetti [6], Carlo Irace [6], Giuseppe Bifulco [1] and Agostino Casapullo [1]

1 Department of Pharmacy, University of Salerno, 84084 Fisciano, Italy; acapuano@unisa.it (A.C.); egazzillo@unisa.it (E.G.); glauro@unisa.it (G.L.); bifulco@unisa.it (G.B.); casapullo@unisa.it (A.C.)
2 PhD Program in Drug Discovery and Development, Department of Pharmacy, University of Salerno,
84084 Fisciano, Italy
3 Department of Biosciences and Territory, University of Molise, C.da Fonte Lappone, 86090 Pesche, Italy
4 Department of Pharmacy, University of Naples "Federico II", Via Domenico Montesano 49, 80131 Naples, Italy; mariavaleria.dauria@unina.it
5 Department of Molecular Medicine and Medical Biotechnology, University of Naples Federico II,
80131 Naples, Italy; mariagrazia.ferraro@unina.it
6 Biochem Lab, Department of Pharmacy, School of Medicine and Surgery, University of Naples "Federico II", Via Domenico Montesano 49, 80131 Naples, Italy; federica.iazzetti@unina.it (F.I.); carlo.irace@unina.it (C.I.)
* Correspondence: gidurso@unisa.it (G.D.); mariagiovanna.chini@unimol.it (M.G.C.)

Figure S1: *The gel from a DARTS experiment stained with Coomassie. The red boxes highlight bands exhibiting increasing intensity, which have been subjected to subsequent analyses.*

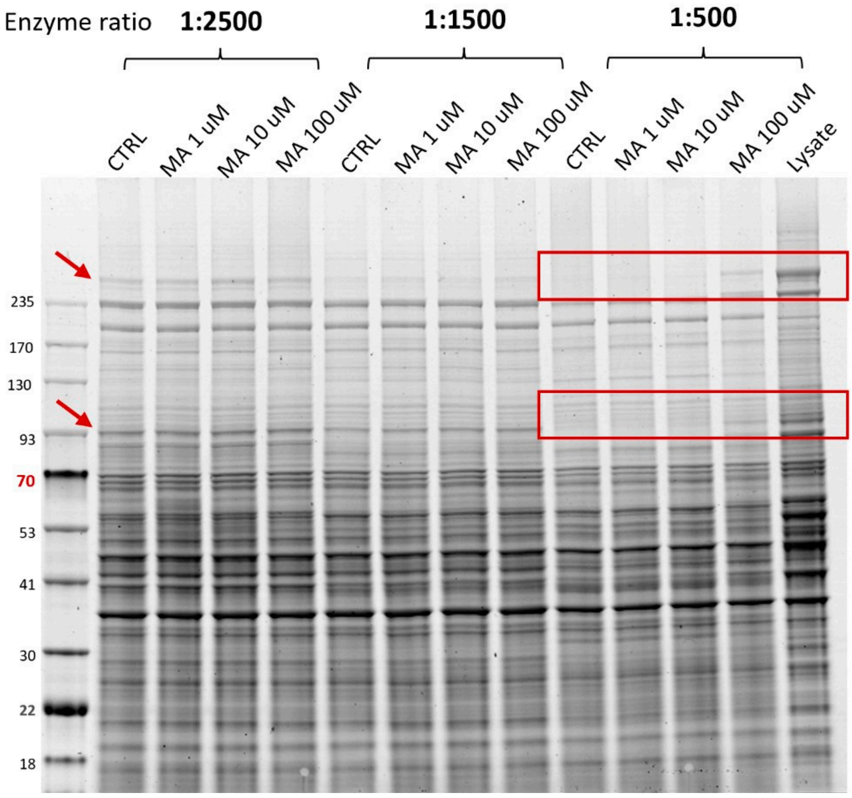

Table S1. *List of MA potential target proteins. The table reports the PD Protection values across replicates expressed as ratios between the abundance in samples treated with different amounts of MA and the control sample. Lys/ctrl is the abundance ratio across the positive control (no MA, undigested) and the negative control (no MA, digested).*

DESCRIPTION	1uM/ctrl	10uM/ctrl	100uM/ctrl	lys/ctrl
Plectin	1.615	2.325	3.352	9.262
Probable ubiquitin carboxyl-terminal hydrolase FAF-X	0.799	0.973	3.98	13.421
Spectrin alpha chain, non-erythrocytic 1	1.374	1.409	2.066	17.188
Spectrin beta chain, non-erythrocytic 1	1.498	1.687	3.214	8.616
Fatty acid synthase	2.517	3.368	5.443	23.426
Protein FAM186A	3.18	4.58	8.336	29.773
CAD protein	2.744	3.493	5.803	17.601
AMP deaminase 2	1.458	2.222	2.671	6.866
Dynein assembly factor 5, axonemal	3.716	3.301	6.243	35.754
RNA cytosine C(5)-methyltransferase NSUN2	0.811	0.671	3.156	12.837
Nucleolin	1.61	2.387	2.096	35.563

Table S2. *Final MRM methods for FAS comprehensive of all the selected transitions. The table shows for each peptide the Q1 and Q3 m/z value and the amino acidic sequence.*

FAS Selected Transitions					
Q1_mz	Q3_mz	ID	Q1_mz	Q3_mz	ID
740.91	752.75	V[1665-1694]R	735.36	869.41	F[1712-1724]R
472.76	559.28	V[1086-1104]R	704.87	548.27	L[2264-2275]R
436.21	261.16	D[1725-1739]K	703.41	708.45	D[2126-2138]R
384.96	262.15	V[1242-1255]	694.82	1002.49	A[2194-2206]K
333.44	201.12	V[1052-1063]R	693.88	903.48	G[1740-1752]K
312.67	312.18	V[2472-2482]R	676.87	231.12	M[1159-1171]R
1031.19	347.19	M[2088-2114]K	676.35	880.5	D[791-802]R
1015.16	361.2	G[478-504]R	675.83	708.39	S[1394-1404]R
947.48	808.42	L[968-992]K	651.84	307.14	A[1132-1142]K
929.20	244.17	A[902-927]K	649.84	514.28	V[299-310]R
906.12	890.46	H[444-468]R	645.79	272.17	V[958-967]R
890.11	829.44	C[1471-1495]K	632.38	242.67	L[374-384]R
852.07	870.45	H[1553-1574]R	625.81	261.09	M[1-12]K
851.10	807.86	T[677-699]K	621.35	415.2	E[1809-1819]R
848.08	868.91	S[275-298]K	616.26	726.3	A[214-224]R
824.77	1009.55	L[803-825]R	596.79	691.31	L[203-213]K
808.11	215.14	T[2483-2505]R	558.34	743.5	S[225-235]K
792.45	242.15	L[1188-1208]K	543.31	828.52	Q[317-326]K
790.03	567.28	G[998-1019]R	539.77	201.12	L[144-151]R
756.03	244.17	D[1977-1995]K	537.27	228.13	Q[2295-2303]R
730.04	936.44	R[1405-1423]R	535.27	272.17	D[1172-1180]R
718.34	851.9	E[612-631]K	522.30	844.49	A[1073-1082]R
717.03	839.41	A[2276-2294]R	521.82	573.37	E[2169-2177]R
706.35	262.15	G[102-122]R	519.83	440.8	G[826-835]K

623.94	504.28	F[1117-1131]R	519.76	517.27	Y[1996-2004]R
610.99	495.3	F[1772-1787]K	506.82	313.19	V[2397-2406]K
593.34	665.35	L[1209-1225]K	498.33	896.58	V[2383-2391]K
562.63	758.39	G[1430-1444]K	479.29	376.22	G[1143-1151]K
546.26	637.29	C[1828-1841]R	478.77	762.45	H[1044-]R
486.61	601.37	G[774-786]K	461.24	371.19	G[469-477]R
474.89	485.25	C[634-646]K	461.24	258.11	Q[2414-2421]R
456.94	620.38	E[1338-1349]R	454.23	288.2	A[2429-2436]K
399.53	200.1	Q[71-80]R	454.23	462.27	W[1546-1552]R
397.55	430.27	S[523-533]K	449.26	204.13	L[1583-1591]K
368.20	316.2	Y[2439-2447]R	440.74	767.39	L[1066-1072]K
272.14	274.12	C[1759-1765]R	440.21	322.19	D[1508-1515]R
958.99	515.25	E[258-274]R	432.73	338.69	G[505-512]R
902.46	288.2	A[1445-1461]R	424.73	229.12	D[1575-1582]K
887.38	593.27	G[2027-2043]R	423.75	242.15	A[42-49]R
857.40	319.17	W[1592-1606]R	414.75	715.41	L[437-443]R
842.93	681.32	V[242-257]K	407.75	315.2	V[1613-1620]K
811.97	426.27	V[1852-1866]K	402.73	563.3	L[2187-2193]K
807.42	515.33	E[2207-2220]R	395.72	335.15	L[1181-11287]R
797.46	213.16	V[884-897]K	387.23	260.2	V[1152-1158]K
775.40	291.17	A[943-957]K	375.22	505.3	L[1872-1878]K
774.87	232.08	A[1226-1239]K	374.20	462.27	G[1539-1545]R

```
     1
5MY0 S--EEVVIAGMSGKLPESENLQEFWANLIGGVDMVTDDDRRWKAGLYGLPKRSGKLKDLSKFDASFFGVHPKQAHTMDPQ
3HHD STGEEVVIAGMSGKLPESENLQEFWDNLIGGVDMVTDDDRRWKAGLYGLPRRSGKLKDLSRFDASFFGVHPKQAHTMDPQ

5MY0 LRLLLEVSYEAIVDGGINPASLRGTNTGVWVGVSGSEASEALSRDPETLLGYSMVGCQRAMMANRLSFFFDFKGPSIALD
3HHD LRLLLEVTYEAIVDGGINPDSLRGTHTGVWVGVSGSETSEALSRDPETLVGYSMVGCQRAMMANRLSFFFDFRGPSIALD

5MY0 TACSSSLLALQNAYQAIRSGECPAALVGGINLLLKPNTSVQFMKLGMLSPDGTCRSFDDSGSGYCRSEAVVAVLLTKKSL
3HHD TACSSSLMALQNAYQAIHSGQCPAAIVGGINVLLKPNTSVQFLRLGMLSPEGTCKAFDTAGNGYCRSEGVVAVLLTKKSL

5MY0 ARRVYATILNAGTNTDGSKEQGVTFPSGEVQEQLICSLYQPAGLAPESLEYIEAHGTGTKVGDPQELNGITRSLCAFRQA
3HHD ARRVYATILNAGTNTDGFKEQGVTFPSGDIQEQLIRSLYQSAGVAPESFEYIEAHGTGTKVGDPQELNGITRALCATRQE

5MY0 PLLIGSTKSNMGHPEPASGLAALTKVLLSLEHGVWAPNLHFHNPNPEIPALLDGRLQVVDRPLPVRGGNVGINSFGFGGS
3HHD PLLIGSTKSNMGHPEPASGLAALAKVLLSLEHGLWAPNLHFHSPNPEIPALLDGRLQVVDQPLPVRGGNVGINSFGFGGS

5MY0 NVHVILQPNTRQAPAPTAHAALPHLLHASGRTLEAVQDLLEQGRQHSQDLAFVSMLNDIAATPTAAMPFRGYTVLGVEGR
3HHD NVHIILRPNTQPPPAPAPHATLPRLLRASGRTPEAVQKLLEQGLRHSQDLAFLSMLNDIAAVPATAMPFRGYAVLGGERG

5MY0 VQEVQQVSTNKRPLWFICSGMGTQWRGMGLSLMRLDSFRESILRSDEAVKPLGVKVSDLLLSTDERTFDDIVHAFVSLTA
3HHD GPEVQQVPAGERPLWFICSGMGTQWRGMGLSLMRLDRFRDSILRSDEAVKPFGLKVSQLLLSTDESTFDDIVHSFVSLTA

5MY0 IQIALIDLLTSVGLKPDGIIGHSLGEVACGYADGCLSQREAVLAAYWRGQCIKDAHLPPGSMAAVGLSWEECKQRCPAGV
3HHD IQIGLIDLLSCMGLRPDGIVGHSLGEVACGYADGCLSQEEAVLAAYWRGQCIKEAHLPPGAMAAVGLSWEECKQRCPPGV

5MY0 VPACHNSEDTVTISGPQAAVNEFVEQLKQEGVFAKEVRTGGLAFHSYFMEGIAPTLLQALKKVIREPRPRSARWLSTSIP
3HHD VPACHNSKDTVTISGPQAPVFEFVEQLRKEGVFAKEVRTGGMAFHSYFMEAIAPPLLQELKKVIREPKPRSARWLSTSIP

5MY0 EAQWQSSLARTSSAEYNVNNLVSPVLFQEALWHIPEHAVVLEIAPHALLQAVLKRGVKSSCTIIPLMKRDHKDNLEFFLT
3HHD EAQWHSSLARTSSAEYNVNNLVSPVLFQEALWHVPEHAVVLEIAPHALLQAVLKRGLKPSCTIIPLMKKDHRDNLEFFLA

5MY0 NLGKVHLTGINVNPNALFPPVEFPAPRGTPLISPHIKWDHSQTWDVPVAEDFPN
3HHD GIGRLHLSGIDANPNALFPPVEFPAPRGTPLISPLIKWDHSLAWDVPAAEDFPN
                                                    852
```

Figure S2. *Sequence alignment of human and murine MAT domain of FAS (PDB codes: 3HHD and 5MY0, respectively). The mismatched residues are highlighted in blue.*

A

IC$_{50}$ (μM) – 48h	MDA-MB-231	MCF-7	MCF-10A	HDFa
Myrianthic acid	50 ± 1	46 ± 2	>50	>50
Ursolic acid	23 ± 5	24 ± 3	>50	>50
Orlistat	19 ± 3	13 ± 4	>50	>50

B

IC$_{50}$ (μM) – 72h	MDA-MB-231	MCF-7	MCF-10A	HDFa
Myrianthic acid	25 ± 3	21 ± 6	>50	>50
Ursolic acid	11 ± 3	9 ± 2	>50	>50
Orlistat	4 ± 2	5 ± 1	>50	>50

Figure S3. *IC_{50} values calculated for the indicated healthy and cancer cell lines (i.e., MCF-10A and HDFa, MDA-MD-231 and MCF-7, respectively), following treatments with myrianthic acid, ursolic acid, and Orlistat. The IC_{50} were determined at two different time points treatment: 48h (A) and 72h (B). For the healthy cell lines, the IC_{50} values remained higher than 50 μM across the two considered time points, suggesting the absence of cytotoxicity of all the tested molecules. Conversely, IC_{50} values in the low micromolar range (<50 μM) were detected following in vitro treatments of cancerous cell lines for both 48 and 72 h.*
